# Supplementary material for: Categorical and phenotypic image synthetic learning as an alternative to federated learning
Source: Nat Commun. 2025 Oct 23;16:9384. doi: 10.1038/s41467-025-64385-z (PMC12550077; doi:10.1038/s41467-025-64385-z)
Supplement: Supplementary file 1 — Supplementary Information [file 41467_2025_64385_MOESM1_ESM.pdf]

SUPPLEMENTAL INFORMATION

Supplementary Note 1: Classification model

The classification model utilized a UNet-based architecture enhanced with residual blocks in both the encoder and decoder. Residual blocks employed Leaky ReLU activation functions and instance normalization, while Mamba modules used the SiLU activation function. Downsampling was achieved using residual blocks with a stride of 2, while upsampling employed nearest-neighbor interpolation. Mamba blocks were incorporated into the encoder path following the residual blocks, as well as in the bottleneck. Skip connections use simple concatenation to combine encoder and decoder feature maps.

The models were trained for 1000 epochs, with 250 training iterations per epoch. Stochastic gradient descent (SGD) with an initial learning rate of 0.01 and Nesterov momentum of 0.99 was used as the optimizer. The learning rate decayed to 0 using a polynomial function. The loss function combined cross-entropy loss and dice loss for robust training. Data augmentation was performed using the "batchgenerators" package, with techniques including mirroring, rotation, scaling, low-resolution simulation, gaussian noise, gaussian blur, brightness, contrast, and gamma augmentation. A 5-fold cross-validation strategy was employed for training. The subject-wise classification was based on averaging the classification probabilities across all tumor voxels, with the class having the highest average probability being assigned as the subject-wise final classification. Table S1.1 details the network configuration and training hyperparameters used in this study.

Table S1. Summary of IDH classification network configurations and training hyperparameters.

**Network configurations**	
Number of stages	6
Downsampling strides	[1, 2, 2, 2, 2, 2]
Convolution kernel sizes	[3, 3, 3, 3, 3, 3]
Number of features	[32, 64, 128, 256, 512, 512]
Number of input channels	4
Number of classes	3 (0: non-tumor voxels, 1: IDH mutated, 2: IDH wild-type)
**Training hyperparameters**	
Patch size	192 x 160
Batch size	110
Number of epochs	1000
Number of batches per epoch	250
Number of validation batches	50
Initial learning rate	0.01
Nesterov momentum	0.99
Learning rate scheduler	Polynomial decay, exponent=0.9

Supplementary Note 2: Federated Learning

For the federated learning (FL) training, we adapted the framework described in Appendix A to implement an FL strategy. Specifically, three IDH classification models were trained simultaneously on three datasets, each utilizing a GPU node. After every 10 local epochs, training was paused, and the local models from the three nodes were collected for aggregation. The federated averaging (FedAvg) algorithm was used to aggregate the model weights from all nodes. The aggregated global model of the current round was then distributed back to the nodes for the next training round. A total of 100 rounds were conducted, ensuring that the total number of training epochs matched between the centralized and FL setups. Federated training employed the same preprocessed data and training hyperparameters as centralized training, ensuring consistency between the centralized and federated models.

Supplementary Note 3: Latent Diffusion Model

A. Autoencoder

The autoencoder model was specifically developed to process high-dimensional multi-contrast MRI data. It includes a variational autoencoder (VAE) with a super-resolution (SR) module within the decoder, enabling efficient encoding, reconstruction, and spatial resolution enhancement of input images. The VAE architecture employs residual blocks as the fundamental units for both the encoder and decoder, with a vanilla attention module included in the attention layers to improve feature representation. Several loss functions were utilized to optimize the VAE model, including pixel-wise loss, perceptual loss, Kullback-Leibler (KL) divergence loss, and discriminator loss. Additional loss components, such as the structural similarity index (SSIM) and gradient losses, were also evaluated but did not significantly enhance image quality.

To further enhance the spatial resolution of the reconstructed images, the decoder was augmented with an SR module based on a standard UNet architecture. The model was trained in two stages: first, the VAE was trained without the SR module, and then the decoder was fine-tuned with the SR module using the pre-trained VAE model. The SR module training leveraged perceptual loss, pixel-wise loss, and SSIM loss to ensure high-quality reconstructions.

The autoencoder model takes inputs and outputs with dimensions of $128 \times 192 \times 192$ (depth × height × width) and five channels, including 4 MRI sequences and the whole tumor mask. The latent space is represented by a compact dimensionality of $8 \times 16 \times 32 \times 32$ (channels × depth × height × width). The model was trained on 4 A100 GPUs using a distributed data-parallel strategy. A batch size of 1 and a 16-bit precision format were adopted to accommodate the computational demands of high-dimensional data. Details of the network architecture and training hyperparameters are presented in Table S2.

Table S2. Summary of the autoencoder network architecture and training hyperparameters.

**VAE model**	
Input/Output Channels	5
Input/Output Dimension	128 × 192 × 192 (D × H × W)
Input/Output Spacing	1.0 × 1.0 × 1.0 mm
Model channels	[128, 256, 512]
Residual Blocks per Stage	2
Attention Type	Vanilla
Dropout Rate	0.1
Latent Space Dimensions	8 × 16 × 32 × 32 (C × D × H × W)
Loss Functions (weights)	Pixel-wise (1.0)<br>Perceptual loss (2.0)<br>KL divergence (1.0e-6)<br>Discriminator (0.5, start after 10001 iterations)
**SR module**	
Number of Stages	4
Downsampling Strides	[1, 2, 2, 2]
Convolution kernel sizes	[3, 3, 3, 3]
Residual Blocks per Stage	[1, 2, 2, 2]
Loss Functions (weights)	Pixel-wise (1.0)<br>Perceptual loss (1.0)<br>SSIM (0.5)
**Training Hyperparameters**	
Batch Size	1
Learning Rate	1.80e-05
Precision	16-bit
Gradient Clipping	1.0
Early Stopping	50 epochs
Learning Rate Scheduler	CosineAnnealingLR
Optimizer	Adam

B. Latent Diffusion Model

The latent diffusion model (LDM) operated in a compressed latent space derived from the autoencoder. It incorporates text-based conditioning through cross-attention mechanisms, enabling the generation of data that adheres to specific conditions or labels, such as IDH mutation status in this study. The UNet backbone configuration and training hyperparameters were carefully selected to ensure stable convergence and optimal performance. The LDM used L1 loss for training and AdamW as an optimizer. Model training was conducted on 4 A100 GPUs using a distributed data-parallel strategy with a batch size of 4 and a 16-bit precision format, optimizing memory usage and computation speed.

Table S3. Configuration and training hyperparameters of the LDM.

**UNet Backbone Configuration**	
Input/Output Channels	8
Latent Space Dimensions	8 × 16 × 32 × 32 (C × D × H × W)
Model channels	[352, 704, 1056, 1408, 1760]
Attention Resolutions (H/W)	[32, 16, 8, 4]
Attention Along Depth Dimension	True
Cross-Attention Dimension	1024
Conditional Encoder	FrozenOpenCLIPEmbedder (freeze)
Conditional Encoder Layer	Penultimate
**Diffusion Process Parameters**	
Noise Schedule	Linear
Timesteps	1,000
**Training Hyperparameters**	
Batch Size	4
Learning Rate	4.0e-05
Precision	16-bit
Accumulate Gradient Batches	2
Optimizer	AdamW

Supplementary Note 4: Membership Inference Attack (MIA) Using SSIM-Based Similarity Analysis

To evaluate the potential privacy leakage risk from synthetic data generated by CATphishing, we conducted a membership inference attack (MIA) using data from the UTSW site. The LDM was trained on 50% of the UTSW dataset (referred to as "members"), while the remaining 50% was held out and used as the validation set ("non-members"). After training, 100 synthetic samples were randomly selected from the LDM output. For the MIA evaluation, we also randomly selected 100 real images from each of the member and non-member sets.

For each real image, we computed its Structural Similarity Index Measure (SSIM) against all 100 synthetic samples. The highest SSIM score (i.e., the maximum similarity) was retained as the representative similarity between each real sample and the synthetic set. This process resulted in two sets of maximum SSIM scores, one for members and one for non-members. We then calculated AUC to assess the ability of these similarity scores to distinguish members from non-members. Figure S1 shows the SSIM score distributions for members and non-members, highlighting substantial overlapping and resulting in an AUC of 0.493, indicating that the SSIM-based MIA could not reliably differentiate between the two groups. These findings suggest that the synthetic images do not retain identifiable features from the training data, supporting the privacy-preserving claims of the CATphishing framework.

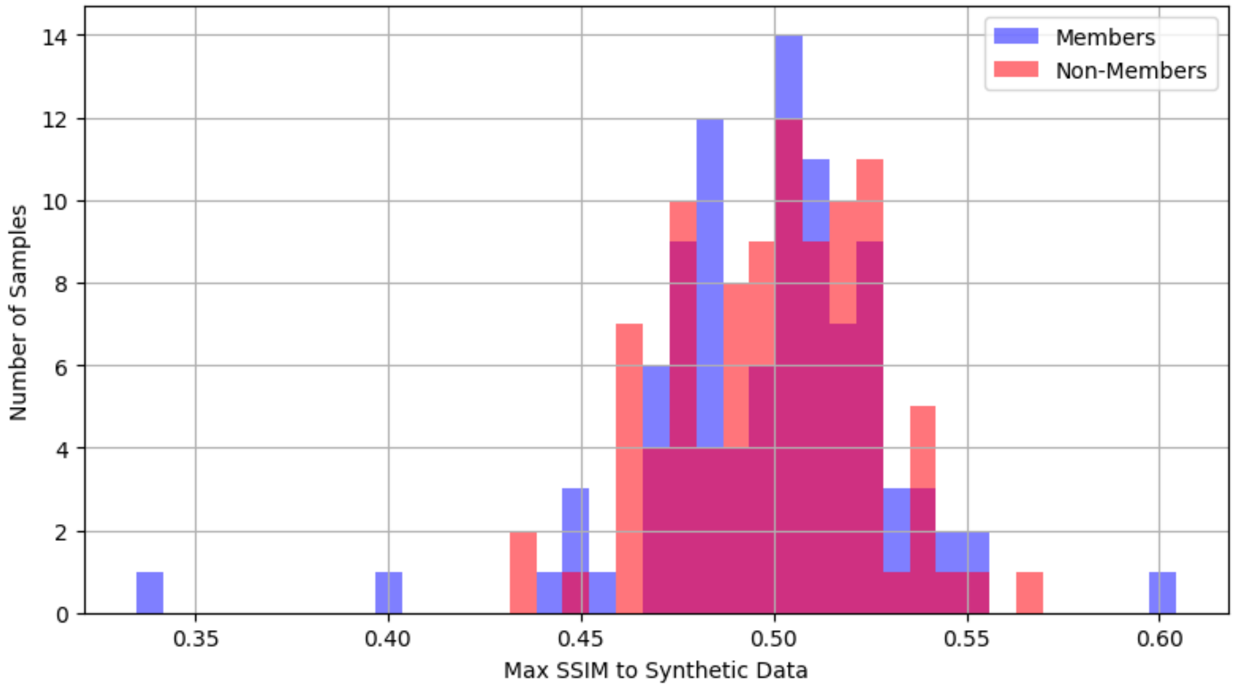

Figure S1. SSIM score distributions between real images (members and non-members) and synthetic images generated by CATphishing. “Members” (blue) were part of the LDM training set; “Non-members” (red) were held out during training. Source data are provided as a Source Data file.

Supplementary Note 5: Supplemental Examples of Synthetic MRI Across Tumor Subtypes

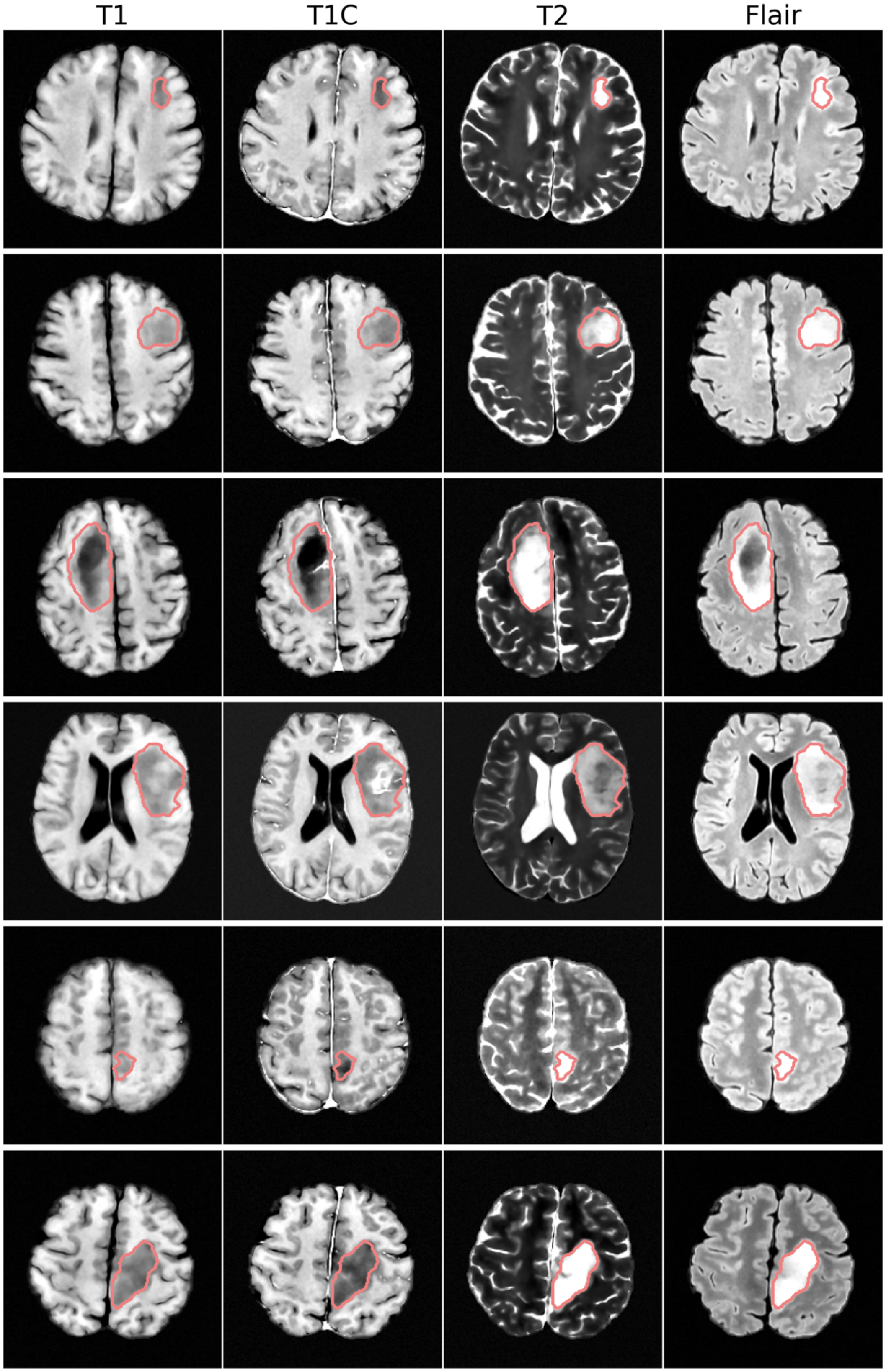

Figure S2. Examples of synthetic MRI for oligodendroglioma (IDH-mutated and 1p19q co-deleted).

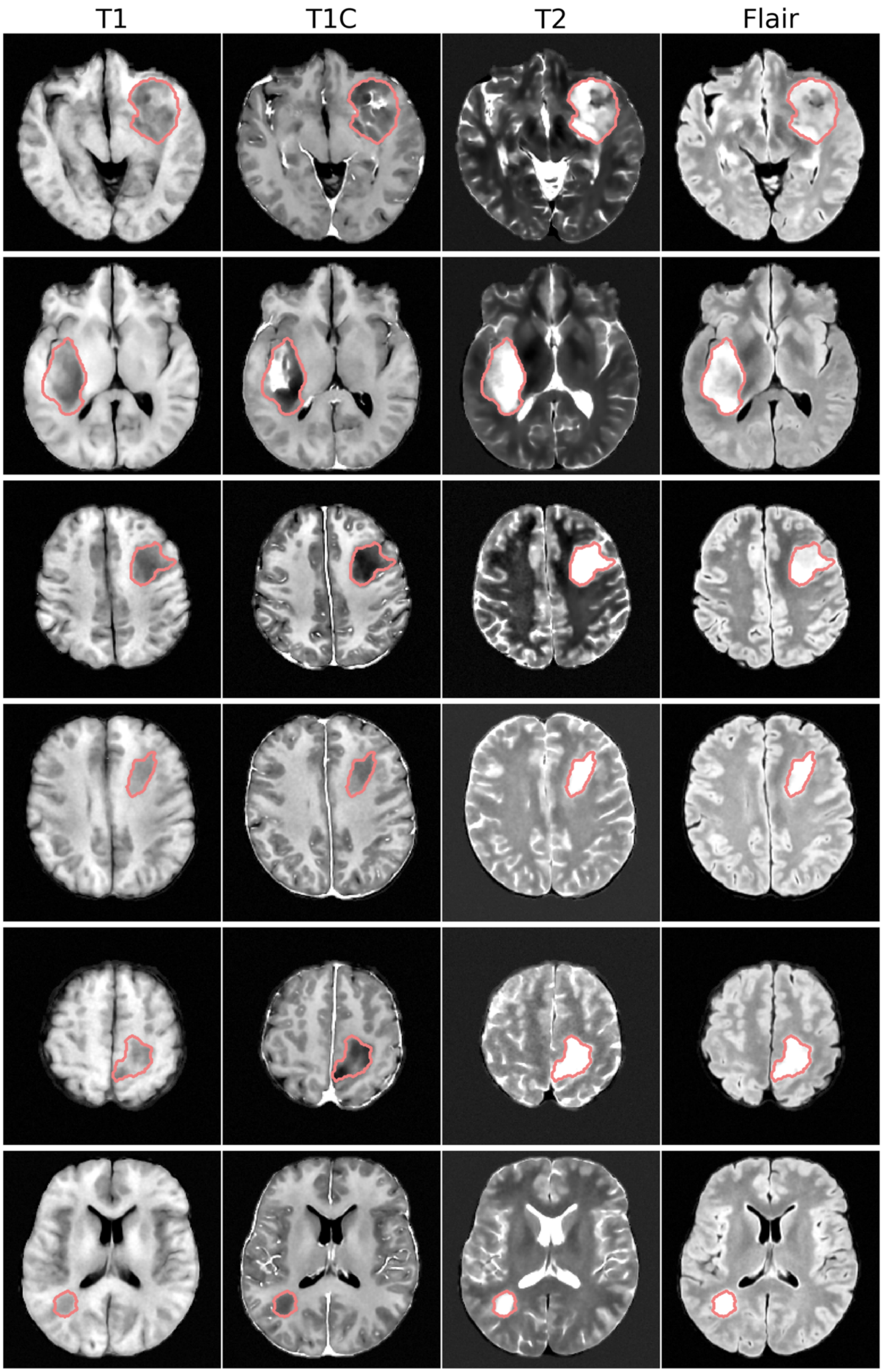

Figure S3. Examples of synthetic MRI for astrocytoma (IDH-mutated and 1p19q non-codeleted).

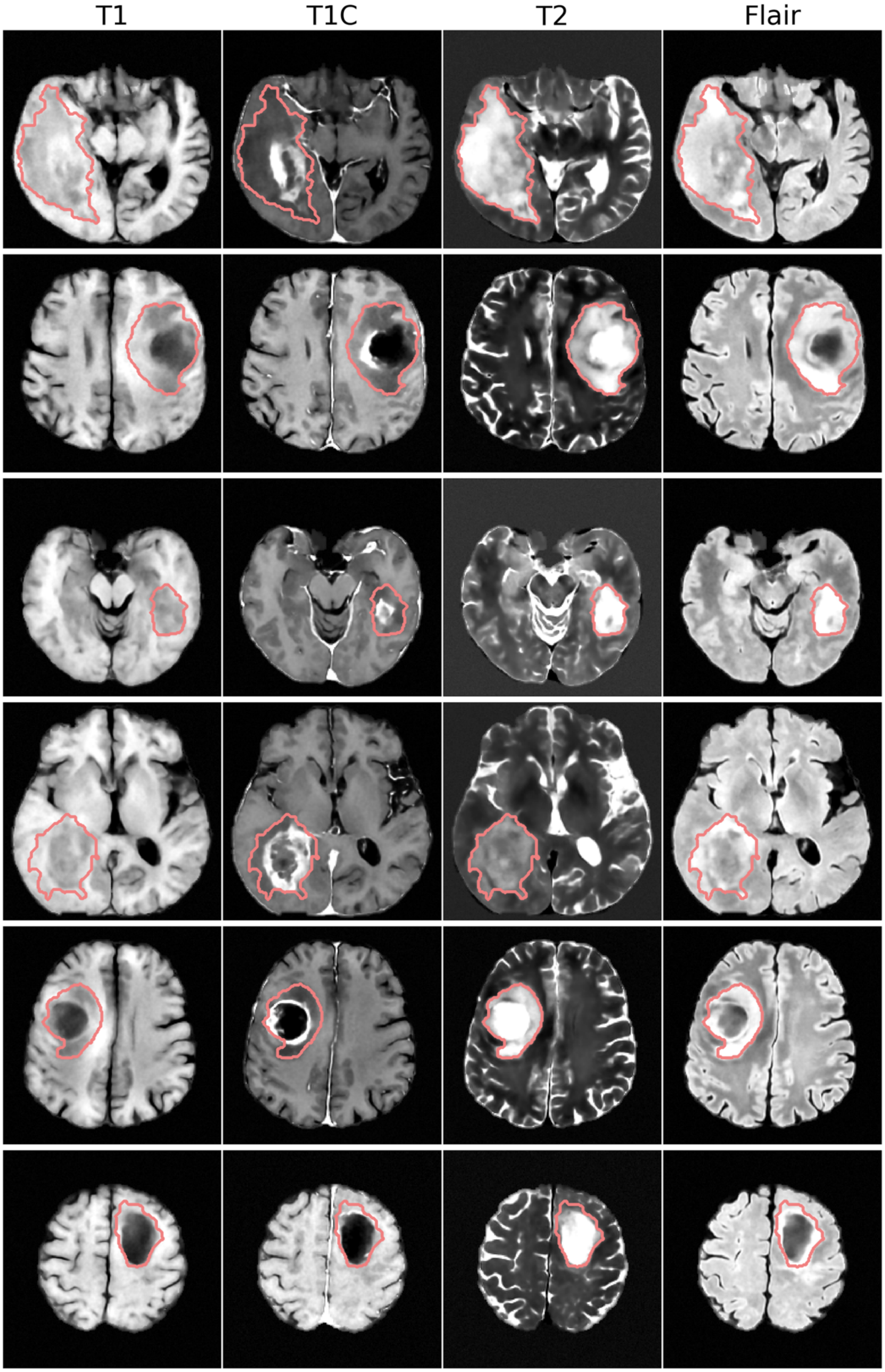

Figure S4. Examples of synthetic MRI for glioblastoma (IDH-wildtype).

Supplementary Note 6: Additional IDH Classification Performance

Table S4 provides a comprehensive evaluation of the first-stage classification model from the two-stage tumor-type classification framework. These results were obtained by testing all cases with available IDH status, while the results in Table 5 were limited to cases with available tumor-type labels. Additionally, the UPenn dataset was included in this evaluation, which was excluded in Table 5 due to the absence of 1p19q status. Overall, all three approaches demonstrated high classification performance across multiple institutions. McNemar's test p-values indicated no statistically significant difference between the performance of the centralized training and FL or CATphishing.

Table S4: Summary of the IDH mutation status classification performance of different models trained on real or synthetic MRI data. Statistical comparisons of classification results among methods are performed using the McNemar test. All tests are two-sided, and no adjustments are applied. Source data are provided as a Source Data file.

Experiments	Training data	Metrics	UCSF	EGD	UWM	UTSWp2	UPenn	**Overall**	McNemar Test
Centralized Training	Real TCGA + Real UTSW + Real NYU	ACC	95.8	96.7	95.4	95.1	97.4	96.3	-
		SEN (MT)	88.4	94.7	83.3	92.0	54.6	90.4	
		SPE (WT)	97.7	97.7	96.5	96.5	98.5	97.7	
		AUC	0.981	0.988	0.960	0.995	0.984	0.985	
FL using the FedAvg algorithm with 100 FL rounds	Real TCGA + Real UTSW + Real NYU	ACC	95.6	96.9	94.5	95.1	97.6	96.2	$\chi^2(1) = 0$, $p = 1$
		SEN (MT)	86.4	95.3	83.3	90.0	63.6	90.1	
		SPE (WT)	98.0	97.7	95.5	97.4	98.5	97.7	
		AUC	0.985	0.990	0.963	0.995	0.982	0.988	
Centralized Training using Synthetic Samples	Synthetic TCGA + Synthetic UTSW + Synthetic NYU	ACC	95.4	95.0	95.9	92.7	96.9	95.4	$\chi^2(1) = 0.706$, $p = 0.4008$
		SEN (MT)	87.4	93.3	88.9	86.0	63.6	89.2	
		SPE (WT)	97.5	95.8	96.5	95.6	97.8	96.9	
		AUC	0.973	0.978	0.957	0.978	0.958	0.975	

Note. Sensitivity (SEN) and specificity (SPE) correspond to the accuracy of the mutated (MT) and wild-type (WT) classes, respectively. ACC = accuracy, AUC = area under the receiver operating characteristic curve, EGD = Erasmus Glioma Database, NYU = New York University, TCGA = The Cancer Genome Atlas, UCSF = University of California San Francisco Preoperative Diffuse Glioma MRI dataset, UPenn = University of Pennsylvania glioblastoma cohort, UTSW = University of Texas Southwestern Medical Center, UWM = University of Wisconsin–Madison, UTSWp2 = University of Texas Southwestern Medical Center part 2.

Supplementary Note 7: Comparative Computational and Communication Overhead of Centralized Training, FL, and CATphishing

Table S5 summarizes the computational and communication overhead associated with three training strategies: centralized training (CT), FL, and CATphishing. CATphishing avoids direct private data pooling and eliminates the need for iterative communication rounds in FL. However, these benefits come with increased local computational burden, as each site has to train the LDMs, which increases local GPU resource requirements.

Table S5. Comparison of the computational and communication overhead of CT, FL, and CATphishing.

**Framework**	**Training Location**	**Local Compute per Site**	**Central Compute**	**Model Parameters (Trainable)**	**Approximate GPU Hours**	**Note**
Centralized Training	Central server only	None	High (1,000 epochs)	Classifier: 33.4M	~ 35 GPU hours	All compute centralized; requires pooling private data from all sites.
FL using the FedAvg algorithm with 100 FL rounds	- Local sites<br>- Central server (aggregation)	High (1,000 epochs per size)	Moderate (aggregation only)	Classifier: 33.4M	~ 105 GPU hours (3 sites) + waiting time for synchronization and aggregation	Requires synchronization every 10 epochs; central server aggregates model weights.
CATphishing	- Local sites (LDM training)<br>- Central server (downstream task)	High (Autoencoder + LDM training)	High (1000 epochs)	LDM: 1.7B<br>Classifier: 33.4M	~ 288 GPU hours for LDM training + 35 GPU hours for the downstream task.	One-time local LDM training; centralized training on synthetic data; no iterative communication needed.
