# Supplementary material for: Categorical and phenotypic image synthetic learning as an alternative to federated learning
Source: Nat Commun. 2025 Oct 23;16:9384. doi: 10.1038/s41467-025-64385-z (PMC12550077; doi:10.1038/s41467-025-64385-z)
Supplement: Supplementary file 3 — Reporting Summary [file 41467_2025_64385_MOESM3_ESM.pdf]

nature portfolio

Corresponding author(s): Nghi Truong, Joseph Maldjian

Last updated by author(s): Aug 18, 2025

Reporting Summary

Nature Portfolio wishes to improve the reproducibility of the work that we publish. This form provides structure for consistency and transparency in reporting. For further information on Nature Portfolio policies, see our Editorial Policies and the Editorial Policy Checklist.

Statistics

For all statistical analyses, confirm that the following items are present in the figure legend, table legend, main text, or Methods section.

n/a	Confirmed	
☐	☒	The exact sample size (*n*) for each experimental group/condition, given as a discrete number and unit of measurement
☐	☒	A statement on whether measurements were taken from distinct samples or whether the same sample was measured repeatedly
☐	☒	The statistical test(s) used AND whether they are one- or two-sided<br>*Only common tests should be described solely by name; describe more complex techniques in the Methods section.*
☒	☐	A description of all covariates tested
☒	☐	A description of any assumptions or corrections, such as tests of normality and adjustment for multiple comparisons
☐	☒	A full description of the statistical parameters including central tendency (e.g. means) or other basic estimates (e.g. regression coefficient) AND variation (e.g. standard deviation) or associated estimates of uncertainty (e.g. confidence intervals)
☐	☒	For null hypothesis testing, the test statistic (e.g. *F*, *t*, *r*) with confidence intervals, effect sizes, degrees of freedom and *P* value noted<br>*Give P values as exact values whenever suitable.*
☒	☐	For Bayesian analysis, information on the choice of priors and Markov chain Monte Carlo settings
☒	☐	For hierarchical and complex designs, identification of the appropriate level for tests and full reporting of outcomes
☒	☐	Estimates of effect sizes (e.g. Cohen's *d*, Pearson's *r*), indicating how they were calculated

Our web collection on statistics for biologists contains articles on many of the points above.

Software and code

Policy information about availability of computer code

Data collection	Except for the UCSF dataset, all datasets were preprocessed using the federated tumor segmentation (FeTS 2.0) tool.
Data analysis	All source code, configuration files, and training scripts used in this study are available in the Code Ocean capsule with DOI: 10.24433/CO.4976589.v1.

For manuscripts utilizing custom algorithms or software that are central to the research but not yet described in published literature, software must be made available to editors and reviewers. We strongly encourage code deposition in a community repository (e.g. GitHub). See the Nature Portfolio guidelines for submitting code & software for further information.

Data

Policy information about availability of data

All manuscripts must include a data availability statement. This statement should provide the following information, where applicable:

- Accession codes, unique identifiers, or web links for publicly available datasets
- A description of any restrictions on data availability
- For clinical datasets or third party data, please ensure that the statement adheres to our policy

This study utilized retrospective MRI scans from four publicly available and three internal datasets.
Public datasets:
- The TCGA dataset is available at https://www.cancer.gov/ccg/research/genome-sequencing/tcga

- The EGD dataset is available at https://xnat.bmia.nl/data/archive/projects/egd
- The University of California San Francisco Preoperative Diffuse Glioma MRI dataset is available at https://www.cancerimagingarchive.net/collection/ucsf-pdgm/
- The University of Pennsylvania glioblastoma cohort is available at https://www.cancerimagingarchive.net/collection/upenn-gbm/
Internal datasets:
- The internal datasets obtained from UT Southwestern Medical Center (UTSW) will eventually be made available on The Cancer Imaging Archive (TCIA).
- The internal datasets from New York University (NYU) and the University of Wisconsin-Madison (UWM) cannot be shared publicly because of institutional and patient privacy regulations.
Trained latent diffusion models to generate synthetic data used in this study will be shared under a data use agreement for research purposes. Requests can be initiated by contacting the corresponding author.
Source data are provided with this paper.

Research involving human participants, their data, or biological material

Policy information about studies with human participants or human data. See also policy information about sex, gender (identity/presentation), and sexual orientation and race, ethnicity and racism.

Reporting on sex and gender	In the study, sex information for each dataset was obtained by self-reporting as documented by the respective data sources. This information was recorded only for descriptive purposes and was not considered in the study design, statistical analyses, or outcome evaluation. The classification models were developed and evaluated without incorporating sex and other demographic variables, and sex information was not used during the training of the generative models. No sex- or gender-based analyses were conducted because the study's primary focus was on technical model development and evaluation rather than on investigating sex- or gender-related impacts.
Reporting on race, ethnicity, or other socially relevant groupings	Such information has not been collected.
Population characteristics	Such information has not been collected.
Recruitment	Such information has not been collected.
Ethics oversight	Institutional Review Board approval was obtained with a waiver of consent for the use of retrospective or public data.

Note that full information on the approval of the study protocol must also be provided in the manuscript.

Field-specific reporting

Please select the one below that is the best fit for your research. If you are not sure, read the appropriate sections before making your selection.

☒ Life sciences ☐ Behavioural & social sciences ☐ Ecological, evolutionary & environmental sciences

For a reference copy of the document with all sections, see nature.com/documents/nr-reporting-summary-flat.pdf

Life sciences study design

All studies must disclose on these points even when the disclosure is negative.

Sample size	This study utilized retrospective MRI scans from four publicly available and three internal datasets. In total, the dataset consisted of 2,491 unique patients from TCGA (n=198), UTSW (n=360), EGD (n=456), NYU (n=181), UWM (n=218), UCSF (n=495), UPenn (n=419), and a second distinct cohort from UTSW (n=164). This sample size was selected to capture heterogeneity across multiple institutions and imaging protocols, supporting the robustness and generalizability of the proposed models.
Data exclusions	Data from the patients that met the following criteria were included in this study: (a) newly diagnosed with glioma; (b) IDH mutation status was available; (c) preoperative MRI scans with T1-weighted, postcontrast T1-weighted, T2-weighted, and T2-weighted fluid-attenuated inversion recovery sequences were available.
Replication	We verified all experiments can be replicated using our source code.
Randomization	Randomization was not applicable as this study utilized retrospective MRI scans from four publicly available and three internal datasets. The dataset was divided into completely independent training and testing cohorts based on their original sources to prevent overlap or bias. No additional randomization was applied because the datasets were already distinct and predefined.
Blinding	Blinding was not applicable to this study as the dataset labels (e.g., IDH mutation status or tumor type status) were determined prior to the analysis and are inherent to the dataset. Furthermore, the study employed automated computational methods (deep learning models), where the training and testing processes were objective and did not involve subjective judgment by investigators. To ensure impartiality, the testing cohort was completely independent from the training cohort, and performance was evaluated using objective metrics such as accuracy and AUC.

Reporting for specific materials, systems and methods

We require information from authors about some types of materials, experimental systems and methods used in many studies. Here, indicate whether each material, system or method listed is relevant to your study. If you are not sure if a list item applies to your research, read the appropriate section before selecting a response.

Materials & experimental systems

n/a	Involved in the study
☒	☐ Antibodies
☒	☐ Eukaryotic cell lines
☒	☐ Palaeontology and archaeology
☒	☐ Animals and other organisms
☒	☐ Clinical data
☒	☐ Dual use research of concern
☒	☐ Plants

Methods

n/a	Involved in the study
☒	☐ ChIP-seq
☒	☐ Flow cytometry
☐	☒ MRI-based neuroimaging

Plants

Seed stocks	N/A
Novel plant genotypes	N/A
Authentication	N/A

Magnetic resonance imaging

Experimental design

Design type	Resting state
Design specifications	N/A
Behavioral performance measures	N/A

Acquisition

Imaging type(s)	Structural
Field strength	3 T, 1.5 T, 1 T, 0.7 T, 0.5 T, 0.3 T
Sequence & imaging parameters	MR images were acquired using different settings.
Area of acquisition	Whole brain

Diffusion MRI ☐ Used ☒ Not used

Preprocessing

Preprocessing software	Except for the UCSF dataset, all datasets were preprocessed using the federated tumor segmentation (FeTS) tool. The pre-processing pipeline included co-registering MRI scans to a template atlas (SRI24), correcting for bias field distortions, and skull stripping. For the TCGA, UTSW, and EGD datasets, the FeTS tool was also used to segment the whole tumor.
Normalization	Z-score normalization was applied to all non-zero voxels for each MRI sequence.
Normalization template	N/A
Noise and artifact removal	N/A
Volume censoring	N/A

Statistical modeling & inference

Model type and settings: N/A

Effect(s) tested: N/A

Specify type of analysis: ☒ Whole brain ☐ ROI-based ☐ Both

Statistic type for inference (See Eklund et al. 2016): N/A

Correction: N/A

Models & analysis

n/a	Involved in the study
☒	☐ Functional and/or effective connectivity
☒	☐ Graph analysis
☐	☒ Multivariate modeling or predictive analysis

Multivariate modeling and predictive analysis: To assess the realism of the synthetic images, we employed the Fréchet Inception Distance (FID) to estimate the distances between feature representations of synthetic and real images. We further assessed the quality of the synthetic MRI images using no-reference image quality assessment metrics: the Blind/Referenceless Image Spatial Quality Evaluator (Brisque) and the Perception-based Image Quality Evaluator (PIQE).
For the classification models, we evaluated the performance using key metrics, including accuracy, sensitivity, specificity, receiver operating characteristic area under the curve (AUC), and the confusion matrix.
